# Effectiveness of Acupuncture in the Treatment of Parkinson's Disease: An Overview of Systematic Reviews

**DOI:** 10.3389/fneur.2020.00917

**Published:** 2020-08-25

**Authors:** Jinke Huang, Xiaohui Qin, Xiaowen Cai, Yong Huang

**Affiliations:** ^1^The Second Clinical Medical College of Guangzhou University of Chinese Medicine, Guangzhou, China; ^2^Department of Neurology, The Second Affiliated Hospital of Guangzhou University of Chinese Medicine (Guangdong Provincial Hospital of Chinese Medicine; Guangdong Provincial Academy of Chinese Medical Sciences), Guangzhou, China; ^3^School of Traditional Chinese Medicine, Southern Medical University, Guangzhou, China

**Keywords:** Parkinson's disease, acupuncture, systematic review, GRADE, AMSTAR-2

## Abstract

**Background:** The effects of acupuncture on Parkinson's disease (PD) outcomes remain unclear. The aim of this overview was to comprehensively evaluate the methodological quality and applicability of the results of systematic reviews (SRs)/meta-analyses (MAs) that examined the use of acupuncture to treat PD.

**Methods:** Eight databases were searched to retrieve SRs/MAs on the use of acupuncture for the treatment of PD. Two reviewers independently screened and extracted the data using the Assessing the Methodological Quality of Systematic Reviews 2 (AMSTAR-2) checklist to evaluate the methodological quality and using the Grading of Recommendations, Assessment, Development, and Evaluation (GRADE) criteria to assess the evidence quality of the included reviews.

**Results:** A total of 11 SRs/MAs were included. According to the AMSTAR-2 checklist results, all included SRs/MAs were rated as very-low-quality studies. The GRADE criteria revealed 20 studies with very-low-quality evidence, 9 with low-quality evidence, 3 with moderate-quality evidence, and 0 with high-quality evidence. Descriptive analysis showed that acupuncture appears to be a clinically effective and safe treatment for PD.

**Conclusions:** The use of acupuncture for the treatment of PD may be clinically effective and safe. This conclusion must be interpreted cautiously due to the generally low methodological quality and low quality of evidence of the included studies.

## Introduction

Parkinson's disease (PD) is an extrapyramidal movement disorder characterized by static tremors, myotonia, bradykinesia, postural instability, and gait difficulty (1). As the second most prevalent neurodegenerative disorder worldwide, it has been reported that 1–2% of the global population >65 years of age is affected by PD (2). It is expected that the prevalence of PD will nearly double by 2030 (3), which will lead to substantial economic pressure for families and society. In addition to motor symptoms such as rigidity and resting tremor, PD patients also have non-motor symptoms such as sleep disorders, hallucinations, and constipation that seriously affect the mental health and quality of life of patients (4).

PD is a complex and slowly progressive neurodegenerative disease associated with multiple risk factors (5). Age and gender, environmental and behavioral factors, and other disease factors (i.e., sleep disturbances, hypertension, and traumatic brain injury) have been shown to be increased risk factors for the development of PD (5, 6). These risk factors are not only crucial to the early diagnosis of PD but also helpful in the development of effective neuroprotection and health care strategies for appropriate populations at risk for PD (5, 7).

In terms of pathology, PD is closely associated with the loss of dopaminergic (DA) neurons in the substantia nigra pars compacta of the brain caused by familial and/or sporadic factors (8). Although the pathogenic mechanism of PD has not been clarified, regardless of the pathogenic mechanism, the pathological features and clinical symptoms of PD are inextricably linked to the reduction in the number of DA neurons. On the study of the mechanism of the occurrence and development of DA neurons' death, researchers have proposed a variety of hypotheses, and the possible mechanisms with high recognition so far are as follows: (a) misfolding and accumulation of α-synuclein. In 1997, researchers discovered that point mutations in α-synuclein can lead to familial PD and that the main component of Lewy bodies associated with the pathogenesis of sporadic PD was also composed of misfolded and aggregated α-synuclein, from which the relationship between genetic factors and PD was first established. α-synuclein can not only activate the immune response within the brain but also activate the proliferation and differentiation of peripheral T cells, achieving immune-inflammatory damage in concert with B cells and microglia and inducing apoptosis in surrounding normal neurons (9). Furthermore, misfolding and aggregation of α-synuclein can lead to a sustained and uncontrolled endoplasmic reticulum stress response, which in turn activates autophagy and apoptotic signaling in cells and causes apoptosis (10). (b) mitochondrial dysfunctions and oxidative stress. Mitochondrial dysfunctions and oxidative stress are important pathogenetic mechanisms leading to the death of DA neurons in PD patients, and reactive oxygen species generated during this process can cause damage to organelles and nucleic acids. Since neuronal cells have higher metabolic activity than other cells, they are very sensitive to insufficient energy production caused by mitochondrial dysfunctions, and their non-renewable attributes also determine the permanence of reactive oxygen species damage (11). Causes of reactive oxygen species generation in PD patients include dopamine metabolism, reduced glutathione levels, disturbed ion levels, and calcium overload (12). Studies have confirmed that there was a large amount of reactive oxygen species in DA neurons of PD patients, which further corroborated the important role of reactive oxygen species in the pathogenesis of PD (13). (c) Neuroinflammation was involved in the pathogenesis and disease progression of PD. Neuroinflammation in the central nervous system is characterized by the activation of astrogliosis and microglia, which produce a large number of inflammatory factors (e.g., IL-6, IL-8, TNF-α, etc.), chemokines, cytokines, and neuromodulins, which produce a destructive effect on the blood–brain barrier while causing damage and apoptosis of surrounding DA neurons (14). Furthermore, gut microbiota, neurotrophic factors (NTF) deficiency, and others have also been shown to be associated with apoptosis and altered morphology and function of DA neurons (15, 16). Moreover, various biomarkers accompanying the pathogenesis of PD (e.g., plasma superoxide dismutase, lipoprotein cholesterol, high-sensitivity C-reactive protein, plasma lipoprotein-associated phospholipase A2, superoxide dismutase, trefoil factor 3, cholinesterase, and homocysteine) are of great value in the diagnosis of PD and disease severity assessment (17–20).

Drug and non-drug treatments for PD have been reported. Clinically approved drug treatments for PD mainly include levodopa, DA receptor agonists, and monoamine oxidase-B inhibitors. The most commonly used drugs are levodopa preparations; however, there are many complications that accompany the use of levodopa (21). Typical adverse events include both DA effects (e.g., dyskinesia, dystonia, freezing of gait, on–off, and wearing-off phenomena) and non-DA effects (e.g., gastrointestinal symptoms, insomnia, and depression) (22). Therefore, based on the pathogenesis of Parkinson's disease, researchers have attempted to seek effective complementary therapies, and the possible treatment ideas are as follows: (a) Enhance protein degradation pathways. Timely removal of excessive α-synuclein in the matrix and extracellular matrix of nerve cells can protect cells from toxicity. For example, PRX002 is an antibody to the C-terminal sequence of α-synuclein, which reduces protein aggregation in the extracellular matrix by specifically binding α-synuclein. The effect of PRX00 against Parkinson's disease has been validated by phase I clinical trials (23). (b) Enhance mitochondrial function. For example, Vitamin B3 can alleviate neurodegenerative diseases by increasing the intracellular content of NAD+/NADH and enhancing the function of the mitochondrial respiratory chain complex (24). (c) Reduce oxidative stress. Glutathione is an important antioxidant in the organism, and increasing the content of glutathione by intravenous injection can play an effective role in treating PD (25). Superoxide radical dismutation was considered as a novel therapeutic strategy for PD (26). Furthermore, for the progressive death of DA neurons, the search for a therapy in which cells can replace or grow into DA neurons has attracted attention, but such a therapy was still in the preliminary research stage and was not promoted in clinical practice (27–29). Gene therapy was also one of the PD treatment strategies, which could use viral vectors to carry target genes into specific brain regions and alleviated PD behavioral disorders through the expression of target genes, but this therapy was still not widely applied (30). Deep brain stimulation in the subthalamic nucleus, subthalamic nucleus, globus pallidus internus, pedunculopontine nucleus, and thalamic complex by an experienced team of experts may be an effective method to treat both motor and non-motor symptoms in patients with PD; however, clinical trials have shown that it may have cognitive and psychiatric side effects (31).

The above therapies have limitations, such as a single target of action, immature technology, adverse effects, and difficult operation. Therefore, many PD patients seek complementary and alternative therapies. Acupuncture, an economical treatment without adverse effects used in China for more than 2000 years, is widely used worldwide for nervous system disorders including PD. Although the mechanisms underlying the effectiveness of acupuncture for PD remain elusive, studies have shown that acupuncture may help to inhibit the accumulation of toxic proteins in neurological diseases, modulate energy supply based on glucose metabolism, and depress neuronal apoptosis, thus exerting a wide range of neuroprotective effects (32). The advantage of acupuncture therapy lies in having either specific target mechanisms of action or multitargets mechanisms of action. A growing number of systematic reviews (SRs) and meta-analyses (MAs) have been published on the clinical effectiveness and safety of using acupuncture for the treatment of PD, but the results have been conflicting and should be studied further. Therefore, we conducted an overview of SRs/MAs to identify and summarize the existing evidence and to systematically determine the clinical effectiveness and safety of using acupuncture to treat PD.

## Methods

We carried out this study based on high-quality methodological overviews (33, 34) and the Cochrane Handbook (35).

### Inclusion and Exclusion Criteria

The inclusion criteria were as follows: (a) study types: SRs/MAs in which the participants were patients with PD and were diagnosed according to any internationally recognized clinical guidelines; (b) intervention: acupuncture therapy (e.g., manual acupuncture, electroacupuncture, or auricular acupuncture) vs. conventional medication (e.g., madopar or levodopa) or acupuncture therapy combined with conventional medication vs. conventional medication alone; (c) outcomes: reviews that assessed the motor and non-motor symptoms of PD as the main outcome measures were considered eligible. Measures of quality of life and activities of daily living were included if they were relevant to the assessment of PD symptoms. Studies were excluded if they used acupuncture alone or combined with other drugs in the control group.

### Search Strategy

We searched the PubMed, Embase, Web of Science, Cochrane Library, China National Knowledge Infrastructure (CNKI), Sino-Med, Chongqing VIP, and Wanfang Data databases from inception to Feb 2020. The keywords used for the search were as follows: Parkinson disease, acupuncture, systematic review, and meta-analysis. The search strategy for the PubMed database is shown in Table 1, and it was adjusted for each database. We also searched conference abstracts and the reference lists of all retrieved articles to avoid missing relevant SRs/MAs.

**Table 1:** Search strategy for the PubMed database.

**Query**	**Search term**
# 1	Parkinson Disease [Mesh]
# 2	Parkinson Disease [Title/Abstract] OR Parkinson's Disease [Title/Abstract] OR Parkinsonism [Title/Abstract] OR tremor syndrome [Title/Abstract] OR Paralysis Agitans [Title/Abstract]
# 3	#1 OR #2
# 4	Acupuncture [Mesh]
# 5	Acupuncture [Title/Abstract] OR pharmacoacupuncture [Title/Abstract] OR acupotomy [Title/Abstract] OR acupotomies [Title/Abstract] OR pharmacopuncture [Title/Abstract] OR needle[Title/Abstract] OR needling [Title/Abstract] OR body-acupuncture [Title/Abstract] OR electroacupuncture [Title/Abstract] OR electro-acupuncture [Title/Abstract] OR auricular acupuncture [Title/Abstract] OR warm needle [Title/Abstract]
# 6	#4 OR #5
# 7	Meta-Analysis as Topic [Mesh]
# 8	Systematic review [Title/Abstract] OR Meta-Analysis [Title/Abstract] OR meta-analyses [Title/Abstract]
# 9	#7 OR #8
# 10	#3 AND #6 AND #9

### Data Extraction

All included SRs/MAs were screened independently by two reviewers (XH-Q and JK-H), and the data from the reviews were validated and extracted according to the predefined assessment criteria for this study. Any disagreements between the reviewers were resolved by discussion or by consulting a third reviewer for a final decision.

### Quality Assessment

Two reviewers (XH-Q and JK-H) independently evaluated the quality of the included articles by using the Assessing the Methodological Quality of Systematic Reviews 2 (AMSTAR-2) checklist (36). Any discrepancies in the ratings of the 16 items from the AMSTAR-2 checklist were resolved by discussion and adjudication by an experienced and authoritative third reviewer (Y-H). The Grading of Recommendation, Assessment, Development, and Evaluation (GRADE) framework (37) was used to categorize the quality of evidence into four levels: high, moderate, low, or very low. The initial grading could be decreased if there are study limitations, inconsistencies, imprecision, indirectness, or publication bias (38). Any disagreements were resolved by consensus or discussion with an experienced and authoritative third reviewer (Y-H).

## Results

### Literature Search

**Figure 1:**
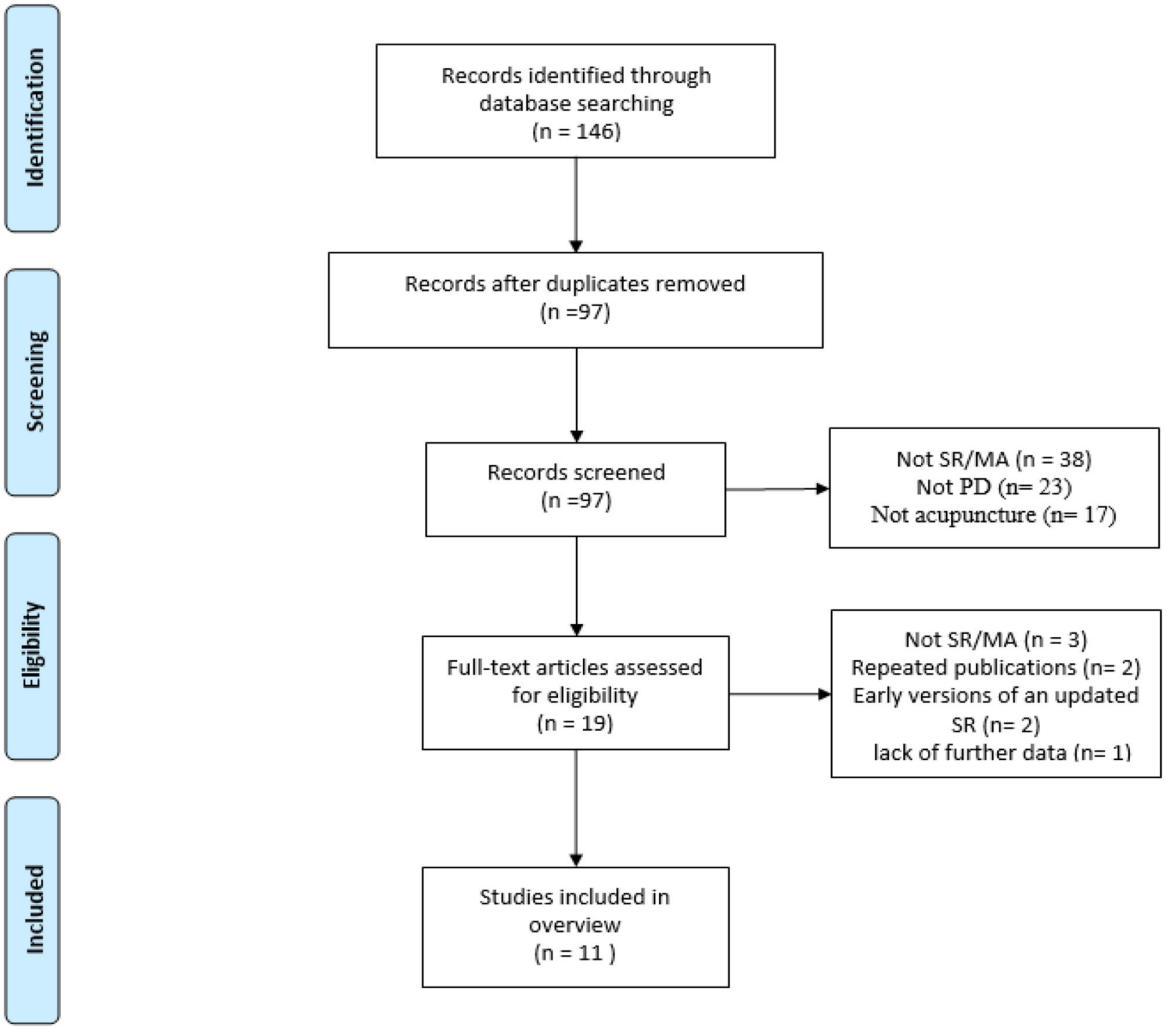
Flowchart of study selection.

Our search identified 146 articles. After screening the titles and abstracts, 127 duplicates and ineligible studies were excluded. The remaining 19 records were considered to be of interest. After full-text review, eight reviews were excluded due to not being SRs/MAs (*n* = 3), being duplicate publications (*n* = 2), being early versions of an updated SR (*n* = 2), and lacking the necessary data (*n* = 1). Thus, 11 reviews (39–49) met the inclusion criteria and were included in the final analysis. Figure 1 outlines the article selection process.

### Characteristics of Included Reviews

The characteristics of the 11 SRs/MAs included in our final analysis are summarized in Table 2. They were published between 2010 and 2019, and 9 of them were published since 2015 (39–43, 45–48). The number of primary studies included in each review ranged from 8 to 42, and the sample sizes ranged from 474 to 2,625. The interventions in the therapy groups were mainly acupuncture or acupuncture plus medication, and the interventions in the control groups were mainly medication and sham placebo acupuncture. The conclusions from these reviews differed: 10 reviews reached the conclusion that acupuncture was effective in relieving PD symptoms, while the remaining 1 (47) reached the opposite conclusion.

**Table 2:** Characteristics of the included reviews.

**Reviews**	**Country**	**Trials (sample size)**	**Treatment intervention**	**Control intervention**	**Quality assessment tool**	**Conclusion summary**
Liu et al. (39)	China	12 (864)	Scalp AT, Scalp AT + medication	Medication	Cochrane criteria	Scalp AT (or combined with medication) treatment was superior to medication alone in improving motor symptoms and activities of daily living in PD patients.
Liu et al. (40)	China	12 (892)	AT, EA, AT + madopar	Madopar	Jadad	Acupuncture had a certain clinical effect on PD—it can relieve the clinical symptoms of PD to some extent and postpone the progression of PD, which improves the quality of life of PD patients.
Ou and Xu (41)	China	21 (1,487)	AT, EA, AT + madopar	Madopar	Jadad	Acupuncture was effective in the treatment of PD and had few adverse reactions, and thus, it may be an effective and safe treatment for PD.
Gui et al. (42)	China	8 (590)	AT + madopar	Madopar	Cochrane criteria	Acupuncture combined with drug treatment of PD was slightly better than drug treatment alone.
Yin et al. (43)	China	9 (655)	AT, AT + medication	Medication	Cochrane criteria	The total effective rate of acupuncture for PD was significantly superior to those of the control group.
Sun and Zhang (44)	China	18 (1,325)	AT, AT+madopar	Madopar	Cochrane criteria	Acupuncture treatment had a certain effect on some non-motor symptoms of PD.
Qiang et al. (45)	China	9 (474)	SEA + medication	Medication	Cochrane criteria	The combination of SEA and medication may be a promising intervention for patients with PD, especially regarding the improvement of motor function.
Liu et al. (46)	China	11 (831)	AT + madopar	Madopar	Cochrane criteria	Acupuncture combined with Madopar appeared, to some extent, to improve clinical effectiveness and safety in the treatment of PD, compared with Madopar alone. This conclusion must be considered cautiously, given that the quality of most of the studies included was low.
Noh et al. (47)	Korean	42 (2,625)	AT, EA, AT + medication	Medication, placebo acupuncture	Cochrane criteria	A combination of electroacupuncture and medication was not significantly more effective than medication alone based on the UPDRS III, the Webster Scale, and the Tension Assessment Scale. The results also failed to show that acupuncture was significantly more effective than placebo acupuncture in total UPDRS.
Lee and Lim (48)	Korean	25 (982)	AT, AT + madopar	Madopar, no treatment	PEDro score	Acupuncture was effective in relieving PD symptoms compared with no treatment and conventional treatment alone, and acupuncture plus conventional treatment had a more significant effect than conventional treatment alone.
Yang et al. (49)	China	13 (832)	AT	Madopar	Jadad	Acupuncture was safe and effective in the treatment of PD. Acupuncture plus Western drugs may be superior to Western drugs alone.

*AT, acupuncture therapy; EA, electroacupuncture; SEA, scalp electroacupuncture*.

### Methodological Quality Results

The AMSTAR-2 assessment showed that all reviews had more than one critical item that was not met and were thus classified as very low quality (Table 3). The methodological limitations arose from three major items: item 2 (all included reviews were unregistered), item 4 (no gray literature search was performed in all included reviews), and item 7 (seven reviews did not assess the possible effect of publication bias). Other details can be found in Table 3.

**Table 3:** Result of the AMSTAR-2 assessments.

**Reviews**	**AMSTAR-2**																**Quality**
	**I1**	**I2**	**I3**	**I4**	**I5**	**I6**	**I7**	**I8**	**I9**	**I10**	**I11**	**I12**	**I13**	**I14**	**I15**	**I16**	
Liu et al. (39)	Y	PY	Y	PY	Y	Y	N	Y	Y	Y	Y	Y	Y	Y	N	Y	VL
Liu et al. (40)	Y	PY	Y	PY	Y	Y	N	Y	Y	N	Y	Y	Y	Y	Y	N	VL
Ou and Xu (41)	Y	PY	Y	PY	Y	Y	N	Y	Y	Y	Y	N	Y	Y	N	Y	VL
Gui et al. (42)	Y	PY	Y	PY	Y	Y	N	Y	Y	N	Y	Y	Y	Y	Y	N	VL
Yin et al. (43)	Y	PY	Y	PY	Y	Y	N	Y	Y	Y	Y	Y	Y	Y	Y	Y	VL
Sun and Zhang (44)	Y	PY	Y	PY	Y	Y	N	Y	Y	Y	Y	Y	N	Y	N	Y	VL
Qiang et al. (45)	Y	PY	Y	Y	Y	Y	N	Y	Y	Y	Y	Y	N	Y	N	Y	VL
Liu et al. (46)	Y	PY	Y	PY	Y	Y	N	Y	Y	Y	Y	Y	Y	Y	Y	Y	VL
Noh et al. (47)	Y	PY	Y	Y	Y	Y	N	Y	Y	Y	Y	Y	Y	Y	N	Y	VL
Lee and Lim (48)	Y	PY	Y	PY	Y	Y	N	Y	Y	Y	Y	Y	Y	Y	N	Y	VL
Yang et al. (49)	Y	PY	Y	PY	Y	Y	N	Y	Y	Y	Y	Y	Y	Y	N	Y	VL

*Y, Yes; PY, partial Yes; N, No; VL, Very low; L, Low; H, High*.

### GRADE Results

The 11 SRs/MAs included 32 outcomes related to the effectiveness of acupuncture for treating PD. The GRADE assessment revealed that no outcomes had high-quality evidence, and only three outcomes provided moderate-quality evidence (Table 4). The evidence was downgraded because of the following limitations: (1) All the original RCTs were of low quality. The bias of randomization, blinding, and allocation concealment decreased the validity of the GRADE approach. (2) For 27 (27/32, 84.4%) outcomes, we downgraded the quality of evidence based on publication bias owing to the incomprehensive literature search as well as the number of RCTs. (3) For 18 (18/32, 56.3%) outcomes, we downgraded the quality of evidence based on imprecision owing to the wide confidence intervals or small number of participants (<300). (4) For 10 (10/32, 31.3%) outcomes, we downgraded the quality of evidence based on inconsistency owing to the high heterogeneity. Other details can be found in Table 4.

**Table 4:** GRADE evaluation results.

**Reviews**	**Interventions**	**Outcomes**	**Limitations**	**Inconsistency**	**Indirectness**	**Imprecision**	**Publication bias**	**Quality**
Liu et al. (39)	Scalp AT + madopar vs. Madopar	Webster scale score	−1^①^	0	0	0	−1^④^	L
		UPDRS score	−1^①^	−1^②^	0	−1^③^	−1^④^	VL
		PDSS score	−1^①^	0	0	−1^③^	−1^④^	VL
Liu et al. (40)	AT + madopar vs. Madopar	Effectiveness	−1^①^	0	0	0	−1^④^	VL
		Webster scale score	−1^①^	−1^②^	0	0	−1^④^	VL
		UPDRS score	−1^①^	0	0	−1^③^	−1^④^	VL
Ou and Xu (41)	AT + madopar vs. Madopar	UPDRS score	−1^①^	−1^②^	0	−1^③^	−1^④^	VL
		Webster scale score	−1^①^	0	0	−1^③^	−1^④^	VL
	AT vs. Madopar	UPDRS score	−1^①^	−1^②^	0	−1^③^	−1^④^	VL
		Webster scale score	−1^①^	0	0	−1^③^	−1^④^	VL
Gui et al. (42)	AT + madopar vs. Madopar	Effectiveness	−1^①^	0	0	0	−1^⑤^	L
Yin et al. (43)	AT + madopar vs. Madopar	Effectiveness	−1^①^	0	0	0	−1^⑤^	L
Sun and Zhang (44)	AT + madopar vs. Madopar	HAMD score	−1^①^	0	0	0	−1^④^	L
		UPDRS I score	−1^①^	−1^②^	0	0	−1^④^	VL
		UPDRS II score	−1^①^	0	0	0	−1^④^	L
		Webster scale score	−1^①^	0	0	0	−1^④^	L
Qiang et al. (45)	SEA + medication vs. Medication	UPDRS score	−1^①^	−1^②^	0	−1^③^	−1^④^	VL
		Webster scale score	−1^①^	0	0	−1^③^	−1^④^	VL
		Effectiveness	−1^①^	0	0	0	0	M
Liu et al. (46)	AT + madopar vs. Madopar	Effectiveness	−1^①^	0	0	0	0	M
		UPDRS score	−1^①^	−1^②^	0	0	−1^④^	VL
		Adverse events	−1^①^	0	0	−1^③^	−1^④^	VL
Noh et al. (47)	AT + medication vs. Medication	UPDRS score	−1^①^	0	0	−1^③^	0	M
	EA + medication vs. Medication	UPDRS score	−1^①^	0	0	−1^③^	0	L
	AT vs. placebo AT	UPDRS score	−1^①^	0	0	−1^③^	0	L
Lee and Lim (48)	AT + madopar vs. Madopar	UPDRS score	−1^①^	0	0	0	−1^④^	L
		Webster scale score	−1^①^	−1^②^	0	−1^③^	−1^④^	VL
		Effectiveness	−1^①^	−1^②^	0	−1^③^	−1^④^	VL
	AT + madopar vs. Madopar	Webster scale score	−1^①^	0	0	−1^③^	−1^④^	VL
		Effectiveness	−1^①^	0	0	−1^③^	−1^④^	VL
	AT vs. no treatment	Webster scale score	−1^①^	0	0	−1^③^	−1^④^	VL
		Effectiveness	−1^①^	0	0	−1^③^	−1^④^	VL
Yang et al. (49)	AT vs. Madopar	Effectiveness	−1^①^	−1^②^	0	−1^③^	−1^④^	VL

VL, Very low; L, Low; M, Moderate; H, High. AT, acupuncture therapy; EA, electroacupuncture; SEA, scalp electroacupuncture; UPDRS, Unified Parkinson's Disease Rating Scale; PDSS, Parkinson's Disease Sleep Scale; HAMD, Hamilton Depression Rating Scale;

①: *The experimental design had a large bias in random, distributive findings or was blind*.

②: *The confidence intervals overlapped less, the P-value for the heterogeneity test was very small, and the I^2^ was larger*.

③: *The confidence interval was not narrow enough*.

④: *Fewer studies were included, and there may have been greater publication bias*.

⑤: *Funnel graph asymmetry*.

## Outcomes

### Effective Rate

A total of seven reviews (40, 42, 43, 45, 46, 48, 49) analyzed the effective rate of acupuncture for PD. In six reviews (40, 42, 43, 45, 46, 48) that used effective rate to compare the effects of acupuncture plus madopar treatment vs. madopar treatment alone, the combined treatment had a significantly greater effect on PD symptoms. Two reviews (48, 49) used effective rate to compare the effects of acupuncture vs. madopar treatment alone. The results of Lee (48) showed that acupuncture treatment had a significant effect on PD symptoms [RR = 1.71, 95% CI (0.99,2.96), *P* = 0.06], while the results of Yang (49) showed that there was no significant difference between treatments [RR = 1.17, 95% CI (0.89, 1.54), *P* = 0.1]. One study (48) used effective rate to compare the effects of acupuncture vs. no treatment and found that acupuncture had a significantly greater effect on PD symptoms [MD = 7.36, 95% CI (5.58, 9.14), *P* < 0.00001].

### UPDRS Score

Eight reviews (39–41, 44–48) compared the effects of acupuncture plus madopar treatment vs. madopar treatment alone using the UPDRS score, and the results showed that the combined treatment had a greater effect than madopar treatment alone. However, one review compared the effects of acupuncture vs. madopar alone and found no significant difference in the UPDRS score between treatments [WMD = −2.55, 95% CI (−11.15, 6.05), *P* = 0.56]. In addition, Noh et al. (47) compared the effects of acupuncture vs. placebo acupuncture and observed no significant effect of acupuncture on PD symptoms [MD = −2.46, 95% CI (−11.36, −6.45), *P* = 0.59].

### The Webster Scale

Six reviews (39–41, 44, 45, 48) used the Webster Scale to compare the effects of acupuncture plus madopar vs. madopar alone and found that the combined treatment had a greater effect than madopar alone. In two reviews (41, 48) that used the Webster Scale to compare the effects of acupuncture vs. madopar, a significant effect of acupuncture on PD symptoms was observed [WMD = −2.50. 95% CI (−2.77, −2.23), *P* < 0.00001; WMD = 3.08, 95% CI (2.81, −3.35), *P* < 0.001]. Lee et al. (48) compared the effects of acupuncture vs. no treatment and observed that acupuncture had a significant effect on PD symptoms [WMD = 7.36, 95% CI (5.58, 9.14), *P* < 0.001].

### Other Outcomes

One study (39) compared the effects of acupuncture plus madopar vs. madopar alone on the HAMD score, and the results showed that combined treatment had a greater effect than madopar alone [SMD = −4.42, 95% CI (−6.44, −2.39), *P* < 0.0001]. Sun et al. (44) compared the effects of acupuncture plus madopar vs. madopar alone on the PDSS score, and the results showed that the combined treatment had a significant effect on PD symptoms [SMD = 2.67, 95% CI (−0.27, 5.60), *P* = 0.08].

### Adverse Events

One review (46) showed that acupuncture combined with madopar in the treatment of PD may not significantly improve dyskinesia but may significantly relieve gastrointestinal reactions, on–off phenomena, and mental disorders.

## Discussion

Neurological diseases can manifest a series of symptoms due to abnormalities in nerve structure, biochemistry, or electrophysiology in the brain, spinal cord, or other nerve (6). Most of them are characterized by progressive neurodegeneration and injury closely related to aging, including PD, Alzheimer's disease (AD), amyotrophic lateral sclerosis, et cetera. Although the pathogenesis of neurodegenerative diseases varies, they still share many common pathogenic features and mechanisms, such as misfolded protein aggregation, neuronal apoptosis, synaptic loss, neurotransmitter abnormalities, et cetera (50, 51).

Acupuncture, an effective and safe treatment method used in China for more than 2000 years, has been widely used worldwide for various neurological diseases. In various models of neurological diseases, peripheral nerve stimulation using acupuncture may have protective effects on neural tissues by increasing expression of NTFs, such as brain-derived NTF and glial-derived NTF, in the central nervous system, especially the brain (52). Furthermore, acupuncture may contribute to recovery from functional impairments following brain damage by encouraging neural stem cell proliferation, which is active at the initial stage of injury, and by further facilitating differentiation (52). Therefore, acupuncture may act as a stimulator to activate peripheral nerves at specific acupoints and induce the expression of various NTFs. Subsequently, NTFs induced by this treatment trigger autocrine or paracrine signaling, which stimulates adult neurogenesis and thus exerts therapeutic effects on functional impairment in neurological disorders (28, 52).

Acupuncture has been shown to be neuroprotective in neurodegenerative disorder such as PD and AD (14, 53). Acupuncture exerts its therapeutic effects by activating and increasing the expression of brain NTFs, including brain-derived NTF and glial-derived NTF. Moreover, acupuncture can stimulate the proliferation and differentiation of NTFs, which in turn activate and promote adult neurogenesis (8). NTF levels are regulated by the therapeutic effects of acupuncture and are associated with enhanced survival, proliferation, and differentiation of NTFs. Acupuncture enhances the expression of NTFs, which may stimulate neurogenesis and thus exert a therapeutic effect on the functional recovery in neurological diseases (50). Therefore, one possible neurophysiological mechanism for the therapeutic effects of acupuncture is to regulate the plasticity of the brain (52). There may also be a synergistic effect when acupuncture was combined with medication, again promoting neurogenesis (52).

With the widespread use of acupuncture as a complementary and alternative therapy for PD, the mechanism of action of acupuncture in the treatment of PD has been extensively studied, and the possible mechanisms of its therapeutic effect for PD are as follows: (a) Neuronal survival. Further mechanism studies have confirmed the neuroprotective effects of acupuncture via the activation of survival pathways of Akt and brain-derived NTF in the substantia nigra region. It was confirmed that acupuncture increased the DA neuronal survival via the nigrostriatal pathway, the expression of DJ-1, and the activities of superoxide dismutase and catalase in the striatum (8). Furthermore, researchers reported that acupuncture can activate the hypothalamic melanin-concentrating hormone biosynthesis and attenuate the reduction of tyrosine hydroxylase in the substantia nigra region (50). (b) Neurotransmitters. Researchers reported that acupuncture was able to improve striatal dopamine levels and enhance dopamine availability, which in turn improved motor function (54). The experiment demonstrated that acupuncture could enhance memory and neuronal density in CA3 and dentate gyrus of PD mouse model, while increasing glutathione peroxidase, and decreasing acetylcholinesterase, monoamine oxidase B, and malondialdehyde in the hippocampus (50). Additionally, proteomic analysis demonstrated that acupuncture reversed a variety of proteins in the lesioned motor cortex of the PD model that may be involved in maintaining the balance of neurotransmitters (55). (c) α-synuclein. Researchers reported that acupuncture stimulation promoted the autophagic clearance of α-synuclein and mammalian target of rapamycin-independent autophagic clearance of aggregation-prone proteins in a PD mouse model (56). (d) Neuroinflammation. Further studies showed that acupuncture suppressed tumor necrosis factor-α (TNF-α) and interleukin-1β (IL-1β) in the striatum and midbrain, while it improved nuclear factor-E2-related factor-2 (Nrf2) and Nrf2-regulated antioxidant enzymes in the PD model. In addition, acupuncture could also play a protective role in cranial nerves by regulating blood pressure and improving cerebral circulation (57, 58). Some of the above mechanisms have also been confirmed by imaging-based research in PD patients (50, 59). Overall, acupuncture, like other neuroprotective agents, is anti-inflammatory, antioxidative, antiapoptotic, and neurotrophic, and the advantage of acupuncture therapy lies in having either specific target mechanisms of action or multitarget mechanisms of action (46).

In this overview, the findings regarding the effectiveness of acupuncture for treating PD were as follows. (a) Eleven SRs/MAs examining the use acupuncture for treating PD were included in this overview, and nine (9/11, 81.82%) of them had been published since 2015, indicating that the scientific interest in using acupuncture for PD patients is increasing. The country with the highest number of included SRs was China (9/11, 81.82%), which is the birthplace of acupuncture. (b) Of the included reviews, 90.9% reached positive conclusions. The effect of acupuncture on relieving PD symptoms seems to be better than that of the effects of control treatments, and acupuncture may significantly relieve side effects (e.g., gastrointestinal reaction and mental disorders). However, most SRs/MAs did not draw firm conclusions due to small sample sizes or their low methodological quality. Further research is needed to provide higher-quality SRs/MAs and RCT-based evidence for the use of acupuncture to treat PD. (c) There is considerable room for addressing the methodological quality of the included SRs/MAs. All included SRs/MAs had multiple critical criteria that were unmet (e.g., being unregistered, lacking a gray literature search). Future studies could avoid these obvious deficiencies to improve methodological quality. (d) There is also considerable room for addressing the methodological quality of RCTs. The GRADE results showed that the evidence quality of all outcomes was downgraded because of the risk of bias. Most of these RCTs did not mention the generation of random sequences in detail; most of them did not specifically describe allocation concealment, and the process of allocation concealment was unclear. Because of the nature of acupuncture, only a small part of the included RCTs mentioned blinding. Well-designed and implemented RCTs are considered gold standards for avoiding the risk of bias (60).

A total of 11 SRs/MAs were included in this overview, and the methodological quality evaluation was very low. By comparing and analyzing the clinical efficacy of acupuncture or acupuncture combined with conventional drugs in the treatment of Parkinson's disease, the clinical effective rate, UPDRS score, Webster Scale score, HAMD score, PDSS score, and the main motor symptoms of Parkinson's disease were analyzed. The results showed that the effective rate, UPDRS score, Webster Scale score, HAMD score, and PDSS score in the treatment group were significantly more improved than those in the control group. These results indicate that acupuncture treatment can improve some motor and non-motor symptoms in patients with Parkinson's disease, which may act by regulating neurotransmitter balance, regulating immunity, reducing oxidative stress, and improving brain electrical function. An analysis of the improvement of specific symptoms of Parkinson's disease revealed that acupuncture significantly improved bradykinesia, myotonia, and postural gait, but there was no significant difference in resting tremor between acupuncture and madopar treatment. This may be related to the fact that acupuncture treatment can only produce corresponding pathophysiological changes in the body through indirect acupoint stimulation and microregulate the internal environment of the lesion circuit but cannot directly supplement reduced dopamine to improve transmitter imbalance. These results are helpful for guiding the selective use of acupuncture as an adjuvant therapy according to different clinical manifestations (61).

### Strengths and Limitations

To our knowledge, our study is the first overview of SRs/MAs on the use of acupuncture for treating PD, and the findings of this overview were based on relatively recent evidence, as 81.82% of the included SRs/MAs were conducted in the past 5 years. However, there are some limitations that need to be considered. First, the methodological quality and evidence quality of the included SRs/MAs were generally low; thus, results based on primary studies should be interpreted with caution. Furthermore, we only searched Chinese and English databases, so SRs/MAs published in other languages that met the inclusion criteria may have been missed.

## Conclusion

Acupuncture may be a promising treatment for patients with PD, and it may be especially effective at improving motor function. This conclusion must be interpreted cautiously, given the generally low methodological quality and low quality of evidence of the included SRs/MAs. Additional studies with rigorous experimental designs and larger sample sizes are needed to verify these results.

## Data Availability Statement

The original contributions presented in the study are included in the article/supplementary material, further inquiries can be directed to the corresponding author/s.

## Author Contributions

JH and XQ designed this study. JH, XQ, and YH performed the data extraction and statistical analysis. JH, XQ, XC, and YH revised and approved the final manuscript. All authors contributed to the article and approved the submitted version.

## Conflict of Interest

The authors declare that the research was conducted in the absence of any commercial or financial relationships that could be construed as a potential conflict of interest.

## Abbreviations

PD: Parkinson's disease
SR: systematic review
MA: meta-analysis
AMSTAR-2: Assessing the Methodological Quality of Systematic Reviews 2
GRADE: Grading of Recommendations, Assessment, Development, and Evaluation
RCTs: randomized clinical trials
CNKI: China National Knowledge Infrastructure
UPDRS: Unified Parkinson's Disease Rating Scale
PDSS: Parkinson's Disease Sleep Scale
HAMD: Hamilton Depression Rating Scale
NTFs: neurotrophic factors
DA: dopaminergic
AD: Alzheimer's disease
TNF-α: tumor necrosis factor-α
IL-1β: interleukin-1
Nrf2: nuclear factor-E2-related factor-2.

## References

[1] Chaudhuri KR Schapira AH. Nonmotor symptoms of Parkinson's disease: dopaminergic pathophysiology and treatment. Lancet Neurol. (2009) 8:464–74. 10.1016/S1474-4422(09)70068-7 19375664

[2] Alves G Forsaa EB Pedersen KF Dreetz Gjerstad M Larsen JP Epidemiology of Parkinson's disease. J Neurol. (2008) (Suppl. 5):18–32. 10.1007/s00415-008-5004-3 18787879

[3] Ascherio A Schwarzschild MA. The epidemiology of Parkinson's disease: risk factors and prevention. Lancet Neurol. (2016) 15:1257–72. 10.1016/S1474-4422(16)30230-7 27751556

[4] DeMaagd G Philip A. Parkinson's disease and its management: part 1: disease entity, risk factors, pathophysiology, clinical presentation, and diagnosis. P T. (2015) 40:504–32. 26236139 PMC4517533

[5] Xie F Gao XY Yang WL Chang ZH Yang XH Wei XB. Advances in the research of risk factors and prodromal biomarkers of Parkinson's disease. ACS Chem Neurosci. (2019) 10:973–90. 10.1021/acschemneuro.8b00520 30590011

[6] Ma L Chan P. Understanding the physiological links between physical frailty and cognitive decline. Aging Dis. (2020) 11:405–18. 10.14336/AD.2019.0521 32257550 PMC7069469

[7] Jia XQ Wang ZJ Yang T Li Y Gao SA Wu GR. Entorhinal cortex atrophy in early, drug-naive Parkinson's disease with mild cognitive impairment. Aging Dis. (2019) 10:1221–32. 10.14336/AD.2018.1116 31788334 PMC6844592

[8] Ko JH Lee H Kim SN Park HJ. Does Acupuncture protect dopamine neurons in Parkinson's disease rodent model? A systematic review and meta-analysis. Front Aging Neurosci. (2019) 11:102. 10.3389/fnagi.2019.00102 31139074 PMC6517785

[9] Zhang JZ Zhou DW Zhang ZP Qu XH Bao KW Lu GH. miR-let-7a suppresses alpha-synuclein-induced microglia microglia inflammation through targeting STAT3 in Parkinson's disease. (2019) 519:740–6. 10.1016/j.bbrc.2019.08.140 31547989

[10] Cóppola-Segovia V Cavarsan C Maia FG Ferraz AC Nakao LS Lima MM. ER stress induced by tunicamycin triggers α-synuclein oligomerization, dopaminergic neurons death and locomotor impairment: a new model of Parkinson's disease. Mol. Neurobiol. (2017) 54:5798–806. 10.1007/s12035-016-0114-x 27660269

[11] McWilliams TG Prescott AR Montava-Garriga L Ball G Singh F Barini E. Basal mitophagy occurs independently of PINK1 in mouse tissues of high metabolic demand. Cell Metab. (2018) 27:439–49.e5. 10.1016/j.cmet.2017.12.008 29337137 PMC5807059

[12] Schapira AH Jenner P Etiology and pathogenesis of Parkinson's disease. Movement Disorders. (2011) 26:1049–55. 10.1002/mds.23732 21626550

[13] Mann VM Cooper JM Krige D Daniel SE Schapira AH Marsden CD. Brain, skeletal muscle and platelet homogenate mitochondrial function in Parkinson's disease. Brain. (1992) 115:333–42. 10.1093/brain/115.2.333 1606472

[14] Lee Y Kim MS Lee J. Neuroprotective strategies to prevent and treat Parkinson's disease based on its pathophysiological mechanism. Arch Pharm Res. (2017) 40:1117–28. 10.1007/s12272-017-0960-8 28952032

[15] Zheng W He R Yan Z Huang Y Huang W Cai Z. Regulation of immune-driven pathogenesis in Parkinson's disease by gut microbiota. Brain Behav Immunity. (2020) 87:890–7. 10.1016/j.bbi.2020.01.009 31931152

[16] Lin CY Hsieh HY Chen CM Wu SR Tsai CH Huang CY. Non-invasive, neuron-specific gene therapy by focused ultrasound-induced blood-brain barrier opening in Parkinson's disease mouse model. J Control Rel. (2016) 235:72–81. 10.1016/j.jconrel.2016.05.052 27235980

[17] Yang W Chang Z Que R Weng G Deng B Wang T. QueContra-directional expression of plasma superoxide dismutase with lipoprotein cholesterol and high-sensitivity C-reactive protein as important markers of Parkinson's disease severity. Front Aging Neurosci. (2020) 12:53. 10.3389/fnagi.2020.00053 32210787 PMC7068795

[18] Zhu S Wei X Yang X Huang Z Chang Z Xie F. Plasma lipoprotein-associated phospholipase A2 and superoxide dismutase are independent predicators of cognitive impairment in cerebral small vessel disease patients: diagnosis and assessment. Aging Dis. (2019) 10:834–46. 10.14336/AD.2019.0304 31440388 PMC6675532

[19] Zou J Chen Z Liang C Fu Y Wei X Lu J. Trefoil factor 3, cholinesterase and homocysteine: potential predictors for Parkinson's disease dementia and vascular parkinsonism dementia in advanced stage. Aging Dis. (2018) 9:51–65. 10.14336/AD.2017.0416 29392081 PMC5772858

[20] Weng R Wei X Yu B Zhu S Yang X Xie F. Combined measurement of plasma cystatin C and low-density lipoprotein cholesterol: a valuable tool for evaluating progressive supranuclear palsy. Parkinsonism Relat Disord. (2018) 52:37–42. 10.1016/j.parkreldis.2018.03.014 29574085

[21] Tambasco N Romoli M Calabresi P. Levodopa in Parkinson's disease: current status and future developments. Curr Neuropharmacol. (2018) 16:1239. 10.2174/1570159X15666170510143821 28494719 PMC6187751

[22] Fahn S Oakes D Shoulson I Kieburtz K Rudolph A Lang A. Levodopa and the progression of Parkinson's disease. N Engl J Med. (2004) 351:2498–508. 10.1056/NEJMoa033447 15590952

[23] Bae EJ Lee HJ Rockenstein E Ho DH Park EB Yang NY. Antibody-aided clearance of extracellular α-synuclein prevents cell-to-cell aggregate transmission. J Neuro. (2012) 32:13454–69. 10.1523/JNEUROSCI.1292-12.2012 23015436 PMC3752153

[24] Williams PA Harder JM Foxworth NE Cochran KE Philip VM Porciatti V. Vitamin B3 modulates mitochondrial vulnerability and prevents glaucoma in aged mice. Science. (2017) 355:756–60. 10.1126/science.aal0092 28209901 PMC5408298

[25] Cunha MP Pazini FL Lieberknecht V Budni J Oliveira Á Rosa JM. MPP-lesioned mice: an experimental model of motor, emotional, memory/learning, and striatal neurochemical dysfunctions. Mol Neurobiol. (2016) 54:1–22. 10.1007/s12035-016-0147-1 27722926

[26] De Lazzari F Bubacco L Whitworth AJ Bisaglia M. Superoxide radical dismutation as new therapeutic strategy in Parkinson's disease. Aging Dis. (2018) 9:716–28. 10.14336/AD.2017.1018 30090659 PMC6065289

[27] Yoo J Lee E Kim HY Youn DH Jung J Kim H. Electromagnetized gold nanoparticles mediate direct lineage reprogramming into induced dopamine neurons *in vivo* for Parkinson's disease therapy. Nat Nanotechnol. (2017) 12:1006–14. 10.1038/nnano.2017.133 28737745

[28] Liu Z Wang X Jiang K Ji X Zhang YA Chen Z. TNFα-induced Up-regulation of Ascl2 affects the differentiation and proliferation of neural stem cells. Aging Dis. (2019) 10:1207–20. 10.14336/AD.2018.1028 31788333 PMC6844591

[29] Tan Y Ke M Huang Z Chong CM Cen X Lu JH. Hydroxyurea facilitates manifestation of disease relevant phenotypes in patients-derived IPSCs-based modeling of late-onset Parkinson's disease. Aging Dis. (2019) 10:1037–48. 10.14336/AD.2018.1216 31595201 PMC6764725

[30] Bartus RT Weinberg MS Samulski RJ. Parkinson's disease gene therapy: success by design meets failure by efficacy. Mol Therap J Am Soc Gene Therap. (2014) 22:487–97. 10.1038/mt.2013.281 24356252 PMC3944322

[31] Zhang C Wang L Hu W Wang T Zhao Y Pan Y. Combined unilateral subthalamic nucleus and contralateral globus pallidus interna deep brain stimulation for treatment of Parkinson disease: a pilot study of symptom-tailored stimulation. Neurosurgery. (2020) nyaa201. 10.1093/neuros/nyaa201 32459849 PMC7666906

[32] Tamtaji OR Naderi Taheri M Notghi F Alipoor R Bouzari R Asemi Z. The effects of acupuncture and electroacupuncture on Parkinson's disease: current status and future perspectives for molecular mechanisms. J Cell Biochem. (2019) 120:12156–66. 10.1002/jcb.28654 30938859

[33] Huang JK Shen M Qin XH Guo WC Li H. Acupuncture for the treatment of tension-type headache: an overview of systematic reviews. Evid Based Complement Alternat Med. (2020) 2020:4262910. 10.1155/2020/4262910 32256645 PMC7106880

[34] Huang JK Qin XH Shen M Huang Y An overview of systematic reviews and meta-analyses on acupuncture for post-stroke aphasia. Eur J Integr Med. (2020) 37:101133 10.1016/j.eujim.2020.101133

[35] Higgins J Green S Collaboration C Cochrane Handbook for Systematic Reviews for Interventions. Cochrane Database of Systematic Reviews. Bristol: The Cochrane Collaboration (2011). p. S38.

[36] Shea BJ Reeves BC Wells G Thuku M Hamel C Moran J AMSTAR-2: a critical appraisal tool for systematic reviews that include randomized or non-randomized studies of healthcare interventions, or both. BMJ. (2017) 358:1 10.1136/bmj.j4008 PMC5833365 28935701

[37] Pollock A Farmer SE Brady MC Langhorne P Mead GE Mehrholz J. An algorithm was developed to assign grade levels of evidence to comparisons within systematic reviews. J Clin Epidemiol. (2015) 70:106–10. 10.1016/j.jclinepi.2015.08.013 26341023 PMC4742519

[38] Norris SL Meerpohl JJ Akl EA Schünemann HJ Gartlehner G Chen Y. The skills and experience of GRADE methodologists can be assessed with a simple tool. J Clin Epidemiol. (2016) 79:150–8. 10.1016/j.jclinepi.2016.07.001 27421684

[39] Liu MH Wang SJ Ma J Weng XY Yuan L A meta-analysis of scalp acupuncture for Parkinson's disease. Shizhen J Tradit Chin Med Res. (2019) 30:2011–4.

[40] Liu HY Chen T Deng YD Zhang B Teng S Cai BC A meta-analysis on the clinical effect of acupuncture for Parkinson disease. J Chin Phys. (2018) 20:16–23. 10.3760/cma.j.issn.1008-1372.2018.01.005

[41] Ou YS Xu W A systematic review of acupuncture for Parkinson's disease. Tradit Chin Med J. (2017) 16:34–8.

[42] Gui L Song FF Cai Y A systematic review and meta-analysis of acupuncture combined with madopar in the treatment of Parkinson's disease. J Tradit Chin Med Univ Hunan. (2016) 36:1384–6.

[43] Yin HN Han C Sun ZR Han B Chang Y. Randomized controlled trials of acupuncture for Parkinson's disease: a systematic review and meta-analysis. J Clin Acupuncture Moxibust. (2016) 32:67–70. 23546633

[44] Sun MX Zhang X A meta-analysis on the therapeutic effect of acupuncture on non-motor symptoms of Parkinson's disease. Acad J Shanghai Univ Tradit Chin Med. (2013) 27:41–8.

[45] Qiang TY Gai C Chai Y Feng WD Ma HJ Zhang Y Combination therapy of scalp electro-acupuncture and medication for the treatment of Parkinson's disease: a systematic review and meta-analysis. J Tradit Chin Med Sci. (2019) 6:26–34. 10.1016/j.jtcms.2019.01.005

[46] Liu HJ Chen LX Zhang Z Geng GZ Chen WJ Dong HQ. Effectiveness and safety of acupuncture combined with madopar for Parkinson's disease: a systematic review with meta-analysis. Acupunct Med. (2017) 35:404–412. 10.1136/acupmed-2016-011342 29180347

[47] Noh H Kwon S Cho SY Jung WS Moon SK Park JM. Effectiveness and safety of acupuncture in the treatment of Parkinson's disease: A systematic review and meta-analysis of randomized controlled trials. Comple Ther Med. (2017) 34:86–103. 10.1016/j.ctim.2017.08.005 28917379

[48] Lee SH Lim S. Clinical effectiveness of acupuncture on Parkinson disease: A PRISMA-compliant systematic review and meta-analysis. Medicine. (2017) 96:e5836. 10.1097/MD.0000000000005836 28099340 PMC5279085

[49] Yang L Du Y Xiong J Liu J Wang YN Li Y. Acupuncture treatment for Parkinson disease: a systematic review. Chin J Evid Based Med. (2010) 10:711–7. 25756963

[50] Guo X Ma T. Effects of acupuncture on neurological disease in clinical- and animal-based research. Front Integr Neurosci. (2019) 13:47. 10.3389/fnint.2019.00047 31543763 PMC6729102

[51] Yang Q Huang Z Luo Y Zheng F Hu Y Liu H. Inhibition of Nwd1 activity attenuates neuronal hyperexcitability and GluN2B phosphorylation in the hippocampus. EBioMedicine. (2019) 47:470–83. 10.1016/j.ebiom.2019.08.050 31474551 PMC6796588

[52] Shin HK Lee SW Choi BT. Modulation of neurogenesis via neurotrophic factors in acupuncture treatments for neurological diseases. Biochem Pharmacol. (2017) 141:132–42. 10.1016/j.bcp.2017.04.029 28461125

[53] Tang YS Xu AP Shao SJ Zhou Y Xiong B Li ZG. Electroacupuncture ameliorates cognitive impairment by inhibiting the JNK signaling pathway in a mouse model of Alzheimer's disease. Front Aging Neurosci. (2020) 12:23. 10.3389/fnagi.2020.00023 32116652 PMC7016202

[54] Wang YY Wang Y Liu JH Wang XM. Electroacupuncture alleviates motor symptoms and up-regulates vesicular glutamatergic transporter 1 expression in the subthalamic nucleus in a unilateral 6-hydroxydopamine-lesioned hemi-Parkinsonian rat model. Neurosci Bull. (2018) 34:476–84. 10.1007/s12264-018-0213-y 29508251 PMC5960449

[55] Li M Li L Wang K Su W Jia J Wang X. The effect of electroacupuncture on proteomic changes in the motor cortex of 6-OHDAParkinsonian rats. Brain Res. (2017) 1673:52–63. 10.1016/j.brainres.2017.07.027 28760440

[56] Tian T Sun Y Wu H Pei J Zhang J Zhang Y. Acupuncture promotes mTOR-independent autophagic clearance of aggregation-prone proteins in mouse brain. Sci. Rep. (2016) 6:19714. 10.1038/srep19714 26792101 PMC4726430

[57] Sun J Ashley J Kellawan J. Can acupuncture treatment of hypertension improve brain health? A mini review. Front Aging Neurosci. (2019) 11:240. 10.3389/fnagi.2019.00240 31572163 PMC6753179

[58] LeWitt PA Kymes S Hauser RA. Parkinson disease and orthostatic hypotension in the elderly: recognition and management of risk factors for falls. Aging Dis. (2020) 11:679–91. 10.14336/AD.2019.0805 32489712 PMC7220277

[59] Chu JS Liu TH Wang KL Han CL Liu YP Michitomo S. The metabolic activity of caudate and prefrontal cortex negatively correlates with the severity of idiopathic Parkinson's disease. Aging Dis. (2019) 10:847–53. 10.14336/AD.2018.0814 31440389 PMC6675526

[60] Moher D Hopewell S Schulz KF Montori V Gøtzsche PC Devereaux PJ. CONSORT 2010 explanation and elaboration: updated guidelines for reporting parallel group randomized trials. BMJ. (2010) 340:c869. 10.1136/bmj.c869 20332511 PMC2844943

[61] Sun Q Wang T Jiang TF Huang P Wang Y Xiao Q. Clinical profile of chinese long-term Parkinson's disease survivors with 10 years of disease duration and beyond. Aging Dis. (2018) 9:8–16. 10.14336/AD.2017.0204 29392077 PMC5772861

